# The Hypnotism Craze and Its Dangers

**Published:** 1890-10

**Authors:** 


ARTICLE II.

### THE HYPNOTISM CRAZE AND ITS DANGERS.

Hypnotism, which is now at the height of fashion, is nothing more than a new edition of the threadbare practice of mesmerism somewhat revised, enlarged and greatly improved. It is strange that the medical profession should go wild over an idea so old. Our grandfathers can tell us more about the mesmerism of their young days than we know of it know under its new name, "hypnotism." The recent agitation was principally indulged in by the French physicians up to a quite late date when the epidemic crossed the Atlantic and pervaded the minds of the more superficial physicians who are fond of bluster and excitement. If we take a retrospective view of the recent past we will find the same element in this country indulging in hypnotism that made such a painful exhibition of themselves over the "Brown-Sequard elixir of life" fraud. At the meeting of the British Medical Association at Leeds, England, last summer, we were annoyed by a couple of supercilious nabobs from Paris who persisted in reading lengthy papers on this subject, much to the disgust of the members of the association. We little expected that our more reasonable American profession would run mad over a practice so well known and so little used. We were mistaken. The folly has fully asserted itself and we are obliged to endure its ephemeral consequence notwithstanding any protest we may make. Like the doctrine of Christian Science there is enough virtue in it to be annoying. In fact it covers about the same ground and accomplishes the same work as the devotees of Christian Science, but in a little different way. There is enough truth in both practices to make them dangerous; the one to the individual who is susceptible to the influence, and the other to life in cases where a pathological condition exists; neither practice being adapted to diseased conditions. Hypnotism or mesmerism presents dangerous phenomena, which concerns the public as well as the subject under the influence, and now that much interest is being manifested in the singular effect of the so-called science, those susceptible to influences of this kind are proportionally easily brought under the effect, just as any hysterical person may be thrown into hysteria by frequent renewal of the exciting cause. There is a peculiarity about any person that can be hypnotized. This peculiarity is confined to the nervous system and is closely related to that developed in hysteria. It is quite probable that hypnotism is but a form of hysteria, and that a case of hysteria can be relieved by hypnotism much more readily than by medicines. We have no doubt but that the Christian Scientist can treat hysteria more successfully than the skillful physician.

There are but few people susceptible of being hypnotized, and those who are can be brought under that state as well by one person as by another, and while hypnotism may be used with advantage in dispelling delusions in hysterical women, and for the cure of hysterical pain and other imaginary ailments, it becomes dangerous in such cases in the hands of unscrupulous persons. The social danger is the one most to be feared. Crimes may be committed under its influence, even the mind may become unbalanced and the patient may go crazy on the subject, just as a person becomes deranged over undue excitement at religious meetings and the like. Fortunately for society the nervous organization of but few individuals, in the aggregate, are susceptible to hypnotism, the influence of Christian Scienc, emind cure, spiritualism, mesmerism, charlatanism and like empiricism.—*K. C. Medical Record.*

